# Mobile Shopping during COVID-19: The Effect of Hedonic Experience on Brand Conspicuousness, Brand Identity and Associated Behavior

**DOI:** 10.3390/ijerph19084894

**Published:** 2022-04-18

**Authors:** Wanjing Jiang, Yao Song

**Affiliations:** 1 Xiangshan Film and Television College, Ningbo University of Finance & Economics, Ningbo 315175, China; jiangwanjing@nbufe.edu.cn; 2 Korean Cultural Strategy Institute, Seoul 100744, Korea; 3 College of Literature and Journalism, Sichuan University, Chengdu 610064, China; 4 Digital Convergence Laboratory of Chinese Cultural Inheritance and Global Communication, Sichuan University, Chengdu 610207, China

**Keywords:** Chinese Generation Z, consumer behavior, brand identity, repurchase intention

## Abstract

COVID-19 has impacted economic and social conditions around the globe. In a post-pandemic world, the labor models have been shifting in favor of working from home and shopping toward online purchasing through mobile devices. The pandemic has, in addition to disrupting the world economy, triggered changes in consumer behavior that require a rethinking of marketing efforts from the consumer’s perspective and a fundamental shift in branding strategies and managerial thinking. This paper expanded the understanding of the mobile consumer behavior of Generation Z consumers in China by examining the changes in their behavior in response to the pandemic. We used a structural equation model (SEM) to show that, in mobile shopping, the hedonic experience has played an essential role in signaling brand conspicuousness and product aesthetics, in turn promoting brand identity and associated behavioral reactions. The paper concludes with a discussion of the implications of these changes for branding identity and brand management.

## 1. Introduction

One of the measures adopted to reduce the transmission of COVID-19 is maintaining social distancing. During the pandemic, the Internet has been a particularly important channel for information [1,2], forcing people to increase their online activities and resulting in, among other things, dramatic growth in online commerce [3,4]. In China, 70% of consumers increased their online shopping for this reason [4]. The ease with which members of Generation Z make use of technologies has had a considerable impact on the e-commerce strategies of companies worldwide [5]. Thus, Chinese members of Generation Z are responsible for a significant portion of internet shopping transactions [4,6,7]. Branding efforts have, accordingly, focused increasingly on efforts to develop a following within this demographic in the specific context of mobile commerce [8].

In recent years, the widespread adoption of smartphones has contributed further to the popularity of online shopping [7,9]. As the number of individual consumers online has increased, researchers have concentrated on identifying factors that influence this kind of shopping [3]. Several studies have analyzed the current trends in mobile shopping based on browsing and purchase data from online retailers, for instance, after launching a mobile app [9,10]. A key aspect of the study of mobile shopping behavior involves identifying factors that may influence purchase intention [11]. The evidence suggests that considerations of efficiency and personal circumstances influence purchase intention as well as the perceived value of mobile shopping [12].

Scholars are also paying attention to consumers’ brand identity, that is, their desire to express their personal identity through brand preferences [13]. This research has taken into account, for instance, the effects of brands on the identity of groups of consumers [14], the distinct meaning of various brands for individuals and groups [15], the aesthetics and perceived uniqueness of brands [16], and consumers’ attitudes toward particular brands [17]. The concept of product aesthetics captures the self-expressive consideration of consumers’ choice of one set of products with which they feel greater affinity than another set [18]. Accordingly, especially strong preferences for self-expression through brand choice are likely to be reflected in strong personal aesthetic preferences [19]. In terms of self-expression, the key factor is a brand’s perceived uniqueness, in that preferences for self-expressive brands tend to correlate with perceptions of brand conspicuousness [20] and, in turn, strengthen the behavioral outcomes of these preferences [17].

Having been born into the digital world, the members of Generation Z have never known life without the Internet or smartphones [21,22]. It is often argued that, as a result, these individuals are more comfortable presenting a public self than the members of earlier generations [6,22,23]. A key driver of the perspectives of Generation Z in this regard has been the use of smartphones to create online identities and narratives of individuals’ lives, a behavior that appeals to their sense of uniqueness and preference for personalized products and services [24,25]. In fact, the desire to manifest individual identity is central to the notion of brand identity for Generation Z users [26].

Smartphones, as agents, allow users to share self-expression and identity but also have inherent attributes, in particular, the values and concepts associated with various brands [27]. However, the research on smartphones and branding has, thus far, paid relatively little attention to the effect of hedonic experience on mobile consumer behavior. To help fill this gap in the research, we here provided fresh insights into consumers’ use of brands to express personal identity [28,29]. Extending the notion of brands as a means of self-expression in various contexts—for instance, as identity signals [13,16,30]—we described shifts in the mobile consumer behavior of members of China’s Generation Z that are likely to persist through and beyond the COVID-19 pandemic. Specifically, we investigated the role of brands in consumers’ drive to engage in self-expression in order to explore (1) the effect of the mobile hedonic experience on perceived aesthetics, brand conspicuousness, and, thereby, brand identity and associated behavioral changes in general and (2) theories regarding the use of brands for self-expression.

**Figure 1:**
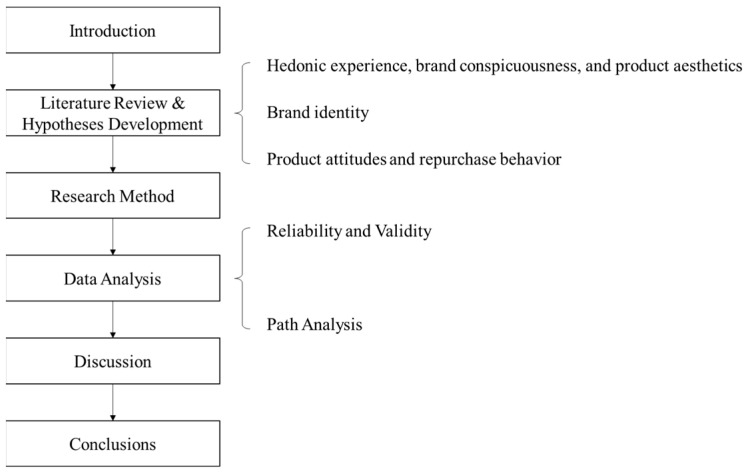
Diagram of the research.

The aim of this study, then, was to develop an integrated model linking hedonic experience to brand conspicuousness, brand identity, and associated behavior in the context of the COVID-19 pandemic, as shown in Figure 1. In what follows, we discuss relevant concepts and theories and describe the development of our hypotheses in Section 2. We explain the research design in Section 3 and, in Section 4, present an analysis of the data indicating that hedonic experience was significantly related to brand conspicuous, perceived aesthetic congruity, and, in turn, brand identity and product attitude. In Section 5 and Section 6, we discuss the results, theoretical contributions, and practical implications of our study.

## 2. Literature Review and Development of Hypotheses

In this section, we present the theoretical framework for the present research, which is summarized in Table 1. The discussion covers the definition and concept of hedonic experience, brand conspicuousness, and product aesthetics in the context of mobile shopping and how it relates to brand identity and associated attitudes and behavior.

### 2.1. Hedonic Experience, Brand Conspicuousness, and Product Aesthetics

Compared with commerce conducted in physical stores, mobile shopping often offers more in the way of diverse product choices, individualized products, service information, and convenience [39]. Consumers’ continued integration of new technologies into their everyday lives has increased their ability to locate and purchase goods and services in a hedonic way, especially through mobile commerce [9].

The extensive literature on hedonic experience has focused particularly on the multisensory, fantasy, and emotive aspects of mobile consumption [32], and the concept continues to evolve. The theory of hedonic experience rests on the notions that consumption is driven by the pleasure that consumers experience using a product and that the criteria for successful consumption are essentially aesthetic in nature [31]. Researchers have attempted to evaluate the impact of such hedonic shopping motives as the desire to be amused, to engage in fantasy, and to experience sensory stimulation [40]. Much of the current literature on hedonic experience suggests that the search—that is, the process of shopping—is often a greater source of pleasure than the actual acquisition of products [32].

Chaudhuri [31] argued that consumers express distinctive identities by seeking a hedonic atmosphere in the shopping experience. That is, they define their self-concept in order to distinguish themselves from other members of the masses [41]. Typically, conspicuous consumption involves lavish spending on brands for the purpose of self-expression through the display of wealth [13]. In other words, some consumers conspicuously use brands as a demonstration of their knowledge of culture, taste, and style [20]. The phenomenon of conspicuous consumption has been the subject of many studies since the publication of Veblen’s *Theory of the Leisure Class* at the turn of the past century [42]. Scholars have begun to examine this form of consumption also among the “lower-upper class” or “nouveaux riches” [43]. This extensive literature focuses particularly on the individual, who is seen as undergoing a motivational process intended to demonstrate elevated social status and an appealing public image [43]. Consumers who engage in conspicuous consumption seem motivated to satisfy such social needs as credibility and prestige as well as material needs [44].

While some scholars have pointed to product attributes as the basis for consumers’ awareness of and opinions about specific products [45], the understanding of the self-expressive function of product attributes is limited. It has been argued that brands strengthen consumers’ intention to purchase by providing information relating to social status [42] based on assessments of the efficacy of perceived conspicuousness, the symbolic role of brands as status symbols, the significance of interpersonal relations, and the potential for upward social mobility expressed through consumption choices [46]. Accordingly, it is possible that, in the context of mobile commerce, hedonic experience promotes brand conspicuousness.

Moreover, consumers pay close attention to the design and look of products along with their functional aspects. Their attentiveness in this regard creates opportunities for manufacturers and marketers to achieve a competitive advantage through the delivery of aesthetic value [47,48]. To analyze the environmental context of consumers’ behavior, researchers have used the stimulus-organism-response (S-O-R) paradigm [49]. This paradigm is based on the notion that environmental cues, especially in hedonic contexts, may elicit certain behavioral responses (e.g., approach or avoidance) by altering subjects’ affect with respect to pleasure, arousal, and perceived aesthetics [49]. Accordingly, we formulated the following hypotheses:

**Hypothesis** **1a** **(H1a).** *Hedonic experience promotes brand conspicuousness in mobile commerce.*

**Hypothesis** **1b** **(H1b).** *Hedonic experience promotes perceived aesthetics in mobile commerce.*

### 2.2. Brand Identity

Brand identity, in the present study, refers to the unique characteristics of brands that consumers use to make distinctions [50,51]. Distinctiveness and prestige tend to strengthen brand identity, making certain brands more attractive to consumers than other [52]. Likewise, brands considered especially distinctive tend to be seen as especially trustworthy, for consumers perceive such brands as more concerned than less distinctive brands to protect their reputations [53] and more likely to fulfill their customers’ expectations [35]. According to social identity theory [54], individuals define themselves as members of various social groups, and brand identity may assist in this sort of social identification [55]. In addition, in the context of contemporary consumerism, which is defined by highly competitive markets, brand identity may help consumers to satisfy attractive and meaningful self-defined needs [35], emphasize their distinctive personalities, and express their values and beliefs [55].

Numerous studies have demonstrated that conspicuous consumption serves to communicate aspects of consumers’ identity to others [56]. Brands hold symbolic value for consumers and feature significantly in the broad spectrum of feelings experienced during the purchase and use of conspicuous products [57]. There is overall agreement that the choice of particular brands may function as a means of communicating aspirations and status [58]. Within social networks, in particular, consumers are often identified based on the products that they possess, and they often assume that other consumers reveal their actual selves in this way [58]. Therefore, consumers tend to choose brands that they perceive to be consistent with their values. Scholars have long debated the nature of the relationship between conspicuous consumption and brand identity. Prior research suggests that, in efforts to assimilate to group preferences with respect to product choices [59], brand identity plays a pivotal role in a range of social interactions and behaviors [60]. We argue that conspicuous consumption is the basis for a brand identity, which functions as an expression of an individual consumer’s social identity. From this perspective, brand identity is an important outcome of the affective commitment to engage in conspicuous consumption.

The perceived aesthetics of products, by extension, could influence consumers’ responses to products and brands [47]. Most of the research on this topic has concerned efforts to make designs attractive so as to enhance product satisfaction, impressions of prestige, and brand value [61]. Regarding specific aesthetic dimensions, researchers have looked for the characteristics of products and brands that elicit positive affections and evaluative responses and thus facilitate customers’ efforts to build brand identity and associated attitudes [33]. Patrick [34], for instance, examined the capacity of everyday aesthetics to enhance consumers’ sense of well-being and attitudes toward products. Other studies have looked at the influence of various design features on the global reception of products and the relative importance of form and function for the evaluation of product attitude [62]. In light of these findings, we formulated a second set of hypotheses:

**Hypothesis** **2a** **(H2a).** *Brand conspicuousness has a positive impact on product attitude.*

**Hypothesis** **2b** **(H2b).** *Brand conspicuousness has a positive impact on brand identity.*

**Hypothesis** **2c** **(H2c).** *Product aesthetics have a positive impact on product attitude.*

**Hypothesis** **2d** **(H2d).** *Product aesthetics have a positive impact on brand identity.*

### 2.3. Product Attitudes and Repurchase Behavior

With regard to consumer behavior, repurchase behavior has been attracting increasing attention. Repurchase intention is an endogenous variable that can be analyzed as an alternative to or in addition to consumers’ actual behavior [63], as explained by Ajzen and Fishbein’s [37] theory of reasoned action. Further, a systematic literature review by Hellier [38] confirmed that positive attitudes toward a particular brand tend to correlate with the intention to purchase or repurchase the brand’s products.

Conceptually, according to the relationship marketing paradigm, repurchase behavior is a significant factor in the long-term survival and profitability of products [50]. Thus, most marketing research has been concerned with repurchase behavior and reducing defections in order to enhance profitability [64,65]. Scholars have proposed various theories regarding the factors that contribute to repurchase. For example, Blut and colleagues [66] showed that brand identity may encourage brand repurchase, and Ahearne [50] analyzed the customer-brand identification relationship and found that brand identity promoted repurchase behavior through product attitude. Based on these considerations, we formulated one more set of hypotheses:

**Hypothesis** **3** **(H3a).** *Brand identity has a positive impact on brand attitude.*

**Hypothesis** **3** **(H3b).** *Brand identity has a positive impact on repurchase intention.*

**Hypothesis** **3** **(H3c).** *Brand attitude has a positive impact on repurchase intention.*

## 3. Research Method

**Figure 2:**
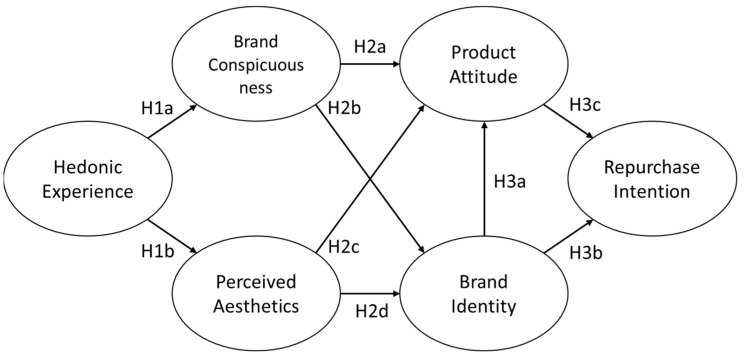
Theoretical framework and hypotheses.

To test our hypotheses, we created a research framework for assessing the effects of hedonic experience on brand conspicuousness, product aesthetics, brand identity, product attitude, and repurchase intention. Figure 2 shows the theoretical framework and nine hypotheses in relation to the variables.

### 3.1. Measurements

We drew the survey questionnaire for this study from those used in similar previous studies, with a five-point, Likert-type scale ranging from “strongly disagree” to “strongly agree” serving to measure the respondents’ attitudes. Our assessment times were retrieved as shown in Table 2. Before collecting the data, we recruited 35 college students to participate in a pre-study to ensure that the questionnaire addressed the research questions effectively. Based on the results of the pre-study, we rephrased questions that were found to be ambiguous, deleted items identified as redundant, and reorganized the structure to enhance concision and clarity [67]. We determined that the resulting survey, which included questions designed to yield demographic information, was appropriate for our purposes.

### 3.2. Sampling and Process

Following the lead of several previous studies [6,21,67,69], we defined members of Generation Z as individuals born in the period from 1995 to 2010 [6]. For purposive sampling that accurately reflected the opinions of members of this generation in China and, therefore, allowed for generalizability of the results, we sampled Chinese college students. Our reasoning was that members of this demographic group tend to be deeply enmeshed in social networks, inhabiting as they do a far more complex media landscape than the members of previous generations and that, to derive value within this setting, many have begun operating as brands themselves. The expanding use and growing social media platforms provide a distribution mechanism such that anyone with access to the technology can become both a creator of content and a broadcaster online. In this environment, Generation Z consumers have become co-creators with various brands as brand identity becomes increasingly participatory and influential in step with the evolution of the affordances of social networks and smartphones in terms of engaging with audiences and expanding their reach [69].

First, we conducted a power analysis with a confidence interval of 0.90, a proportion of 0.5, and a margin of error of 0.05 to estimate the appropriate sample size given the population of Generation Z individuals in Zhejiang Province, which was the setting for our study [70]. The results indicated that a sample of 271 participants would be sufficient for our purposes. Identifying most current college students as members of Generation Z, we conducted a survey of those at major universities in Zhejiang Province in July and August 2021. We chose this setting because, in the 1970s, Zhejiang was an average Chinese province in terms of per capita gross domestic product while today it ranks near the top (specifically, fourth after Beijing, Tianjin, and Shanghai) [71]. Thus, in the era of China’s reform and opening up, the economic development in Zhejiang has followed this strategy [72]. The province’s unique model of economic development is renowned for its remarkable results. Therefore, we expected that consumers in the developed regions would tend to demonstrate more obvious brand preference than those in less-developed regions [20,73].

For the survey, we obtained a list of students from the academic secretaries of each of the universities in the province and then sent a total of 496 survey questionnaires at random to individual college students through their counselors through QQ and WeChat. We received a total of 293 valid responses for a response rate of 59.1%. Appendix A presents the demographics of the sample.

## 4. Data Analysis

We analyzed the data using SPSS 23 and AMOS 24 [74]: SPSS served to analyze the demographic information and reliability, and we relied on AMOS for the confirmatory factor analysis (CFA) and structural equation model (SEM).

### 4.1. Reliability and Validity

We conducted reliability and validity tests before the path analysis to examine the measurement constructs. As Table 3 shows, the results achieved adequate reliability and validity [75]. Specifically, the minimum t-value of the results (9.963) exceeded the threshold of “2”, the minimum Cronbach’s alpha value (0.768) exceeded the threshold of “0.7”, and the minimum standardized factor loading (0.693) exceeded the threshold of “0.5”. Accordingly, the lowest AVE that we calculated (0.525) exceeded the threshold (again, “0.5”).

Table 4 shows the maximum shared variance (MSV) and average shared variance (ASV), which serve to indicate the discriminant validity (threshold: MSV < AVE and ASV < AVE). The results indicate that the model achieved adequate discriminant validity.

Regarding goodness of fit, as Table 5 shows, the standardized root means square residual (SRMR), goodness-of-fit index (GFI and AGFI), root mean square error of approximation (RMSEA), normed fit index (NFI), incremental fit index (IFI), Tucker–Lewis index (TLI), and comparative fit index (CFI) values were all within their respective thresholds, indicating that the current framework achieved an adequate model fit.

### 4.2. Path Analysis

**Figure 3:**
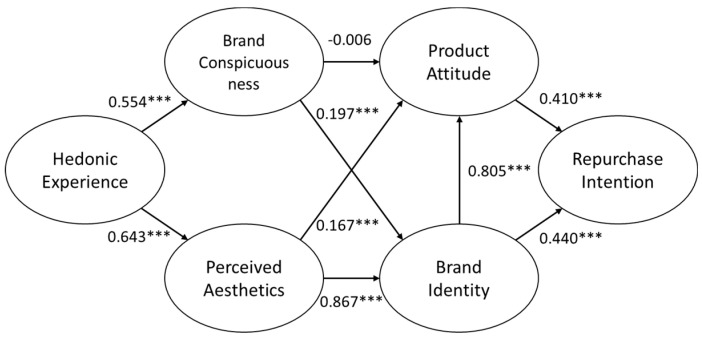
Path analysis for the structural equation model (SEM). Note: *** *p* < 0.01.

Figure 3 and Table 6 summarize the results of the standard coefficients and test results for the hypotheses in order to represent the path analysis.

## 5. Results and Discussion

In this study, we examined the effects of hedonic experience on brand conspicuousness, product aesthetics, brand identity, and associated behavior reactions in the specific context of mobile shopping during the COVID-19 pandemic. We found that hedonic experience played an essential role in signaling brand conspicuousness and product aesthetics, which in turn promoted brand identity and associated behavioral reactions.

The findings presented here contribute to the understanding of the role of hedonic experience, product attitude, brand conspicuousness, and brand identity in mobile shopping. In the first place, though previous research showing that brand attitude positively influenced behavioral intention has attracted considerable interest, the focus of this interest has been on offline consumption. Thus, for instance, several recent studies [25,76,77] suggested that consumers are susceptible to online information and cues related to social identification that could influence purchase behavior, but the authors discussed the findings from the limited perspective of consumers’ social identification, which is to say, without reference to the specific effects of product attitude and brand identity. In designing the present study, we took into account the results of earlier research into online consumer behaviors. We found that brand identity did, in fact, influence repurchase intention and subsequent assessments of value.

From the perspective of the theory of consumer behavior, we shed new light on the effectiveness of purchase intention in relation to hedonic experience with particular attention to brand conspicuousness and brand identity [78,79]. As expected, a favorable product attitude encouraged the repeated purchase of a brand’s products [80]. As individuals advance in age and in their careers, they usually become more likely to purchase known brands. The members of Generation Z tend to prefer communication through social media platforms accessed on various electronic devices and, likewise, to conduct much or even most of their shopping online [80]. Moreover, because these consumers tend to be well-informed and eager to acquire new information and control their lives and futures, they tend to be much less loyal to retailers than, for instance, Millennials [80]. Thus, this study contributes to the literature on repurchase intention by offering preliminary evidence for the impact of brand conspicuousness and brand identity on repurchase behavior [81,82].

We focused on Generation Z because its members have typically been especially prone to establish brand identity in terms of such attributes of products such as aesthetics, consistency with personal taste and style, pleasure, happiness, and/or uniqueness [82,83]. For members of Generation Z in particular, self-perception is determined in part by the groups to which they belong and with which they identify [83]. Several scholars [25,32,76] have pointed out that these consumers have had access to a wide range of options to satisfy their needs through sharing and showing their lives. In addition, when choosing products, members of this generation rely especially heavily on design and aesthetics, regardless of the type of retailer, physical or online, from which they are making a purchase [82]. Among the world’s countries, China has the largest population of members of Generation Z [21]. Most are likely to achieve higher levels of education and income than the members of previous generations and are thus more likely to consume brands with established international reputations. Aligned with the earlier research on which it built, the results of the present study are sufficiently robust to be generalized to other regions such as Japan, Korea, and Thailand [84]. One way in which future researchers could build on these findings is by testing the theoretical framework via conducting surveys in other developing countries and performing cross-national comparisons to contribute to a universal theory of branding [73]. In addition, as mentioned, work is needed to develop multidimensional scales for measuring brand identification in isolation from other factors. Therefore, marketers have opportunities to use hedonic or aesthetic appeals in their strategies. Furthermore, this study provides insights that marketing managers for multinational brands can leverage to enhance their engagement with this new generation of digital native customers.

We acknowledge that the research presented here is subject to certain limitations. To begin with, regarding the methodology, the number of participants in the survey was relatively small and included only relatively young Chinese consumers. Increasing the number of participants and including consumers from other countries would yield more diverse perceptions of and deeper insights into brands and mobile shopping. Further, we did not take into account the impact of the latest mobile technology on the evolution of consumers’ mobile shopping behavior. In addition, the gender ratio was not balanced. Prior research has suggested that gender as well as age may exert less influence on consumers’ attitudes and reactions than has generally been reported [85]. Accordingly, a more balanced gender and age sample would increase the generalizability of future similar studies. Last, though a pilot study was introduced to keep the clarity and consistency of surveys, the adaptation of the scales could potentially influence the validity of the current measurements. Future studies should try to revalidate the current findings.

## 6. Conclusions

With the expansion of mobile technologies, online customers have been enjoying and, indeed, demanding a wide range of choices at highly competitive prices when selecting products and services. Therefore, retailers are facing increasing pressure to ensure that the mobile shopping experience is pleasurable—even as consumers become increasingly critical, less brand-loyal, and, generally, harder to please. In this sense, the insights into mobile shopping, hedonic shopping experience, brand identity, and consumer behavior offered here can help marketers to understand the conspicuous consumption of brands and identify features that customers value, areas for improvement, especially under circumstances, such as the COVID-19 pandemic, that encourage online commerce. Our findings provide a starting point for improving the effectiveness of online retailing through an emphasis on hedonic experience. Thus, we found that hedonic experience played an essential role in signaling brand conspicuousness and product aesthetics, which, in turn, promote brand identity and behaviors associated with it.

## Figures and Tables

**Table 1:** Theoretical Background.

Theory	Scholar(s)	Conclusions
Hedonic experience	H.R. Chaudhuri	Pleasure (i.e., its multisensory, fantasy, and emotive aspects) increases brand conspicuousness and aesthetics.
	H.R. Chaudhuri, et al. [31,32].	
Perceived aesthetics	H.T. Keh [33]	Perceived aesthetics enhance the capacity to build brand identity and product attitude.
	M. Hingle, et al. [34].	
Brand conspicuousness	L. Harris, et al. [35].	Brand conspicuousness helps consumers to satisfy self-defined needs relating to attractiveness and meaning, product attitude, and identity.
	H. He, et al. [36].	
Attitude and behavior	Ajzen [37].	A positive attitude toward a particular brand and brand identity tends to strengthen the intention to repurchase.
	P.K. Hellier, et al. [38].	

**Table 2:** Measurement items of the research.

Measures	Measure Items	Reference
Brand Conspicuousness (BC)	When people use high-end brands, they are more likely to be recognized by others.	Patsiaouras and Fitchett [46]
	I think people who buy high-end brands are more likely to be socially successful.	
	I might envy people who buy high-end brands.	
Hedonic Experience (HE)	Shopping and browsing in online stores is a pleasant pastime for me.	Chaudhuri and Majumdar [32]
	I spend lots of time researching online products because I am interested in mobile shopping.	
	When I shop for products, I like to browse online malls.	
Perceived Aesthetics (AE)	I think the design of mobile shopping fits my aesthetic.	Hagtvedt and Patrick [61]
	I think mobile shopping is very stylish.	
	I think the mobile mall is very attractive.	
Repurchase Intention (RI)	I have purchased online products in the past 2 years.	Wen et al. [63,68].
	I have a high probability of purchasing online products in the next two years.	
	I’m very much looking forward to continuing mobile shopping.	
Product Attitudes (PA)	I think online products are good.	Wilkie and Pessemier [68]
	I think online products are desirable.	
	I think online products are pleasant.	
Brand Identity (BI)	I think the online brands and my image are consistent.	Rather [52]
	I think the online brands and my values are in line.	
	I strongly agree with the online brands.	
	Choosing an online brand makes me feel more innovative in my life	
	Choosing an online brand makes me feel that I am living a healthier life.	

**Table 3:** Reliability and validity of the research.

Factors	Cronbach’s Alpha	Variable	Standardized	*t*-Value	SMC	AVE	Composite
			Factor Loading				Reliability
Brand Conspicuousness (BC)	0.862	BC1	0.851	-	0.724	0.683	0.865
		BC2	0.869	16.176	0.755		
		BC3	0.755	14.151	0.57		
Hedonic Experience (HE)	0.768	HE1	0.711	-	0.506	0.525	0.768
		HE2	0.754	9.963	0.568		
		HE3	0.693	10.334	0.48		
Perceived Aesthetics (AE)	0.926	AE1	0.905	-	0.817	0.808	0.927
		AE2	0.956	22.738	0.818		
		AE3	0.901	22.752	0.791		
Repurchase Intention (RI)	0.924	RI1	0.831	-	0.69	0.818	0.931
		RI2	0.915	20.593	0.837		
		RI3	0.961	22.172	0.923		
Product Attitudes (PA)	0.918	PA1	0.897	-	0.805	0.792	0.919
		PA2	0.869	21.684	0.755		
		PA3	0.898	23.407	0.807		
Brand Identity (BI)	0.951	BI1	0.859	-	0.738	0.952	0.799
		BI2	0.873	28.407	0.762		
		BI3	0.932	24.718	0.868		
		BI4	0.918	23.383	0.842		
		BI5	0.877	24.424	0.769		

Note: Statistical theoretical values are considered according to Gârdan et al.’s work [75].

**Table 4:** Correlation and discriminant validity of the constructs.

	CR	AVE	MSV	ASV	RI	BC	HE	AE	BI	PA
RI	0.931	0.819	0.677	0.429	0.905					
BC	0.865	0.682	0.268	0.220	0.454 ***	0.826				
HE	0.768	0.525	0.394	0.262	0.417 ***	0.518 ***	0.725			
AE	0.927	0.808	0.615	0.439	0.642 ***	0.432 ***	0.628 ***	0.899		
BI	0.952	0.799	0.863	0.523	0.822 ***	0.481 ***	0.512 ***	0.765 ***	0.894	
PA	0.919	0.792	0.863	0.514	0.823 ***	0.453 ***	0.459 ***	0.784 ***	0.929 ***	0.890

Note: *** *p* < 0.01.

**Table 5:** The goodness of fit for the model.

Category	Measure	Acceptable Values	Value
Absolute fit indices	Chi-square		353.893
	d.f.		161
	Chi-square/d.f.	1–5	2.198
	GFI	≥0.80	0.886
	AGFI	≥0.90	0.852
	RMSEA	0.05–0.08	0.064
Incremental fit indices	NFI	≥0.90	0.938
	IFI	≥0.90	0.965
	TLI	≥0.90	0.956
	CFI	≥0.90	0.965

**Table 6:** Results of the path analysis and testing of the hypotheses.

	Path Direction	Standardized Coefficient	Standard Error	C.R. (*t*-Value)	Result
H1a	HE > BC	0.554 ***	0.109	7.317	Accepted
H1b	HE > PA	0.643 ***	0.105	8.840	Accepted
H2a	BC > PA	−0.006	0.038	0.180	Rejected
H2b	BC > BI	0.197 ***	0.043	4.106	Accepted
H2c	AE > PA	0.167 **	0.055	3.353	Accepted
H2d	AE > BI	0.867 ***	0.050	12.734	Accepted
H3a	BI > PA	0.805 ***	0.074	13.183	Accepted
H3b	BI > RI	0.440 ***	0.199	3.267	Accepted
H3c	PA > RI	0.410 ***	0.168	3.026	Accepted

Note: ** *p* < 0.05, *** *p* < 0.01.

## Data Availability

The dataset used in this research are available upon request from the corresponding author.

## Appendix A

**Table A1:** Demographic information for the participants.

Attributes	Value	Frequency	Percentage (%)
Gender	Male	96	32.76%
	Female	197	67.24%
Age	0~18	1	0.34%
	18~25	157	53.58%
	25~40	120	40.96%
	40+	15	5.12%
Education	High School	10	3.41%
	Bachelor	189	64.51%
	Master	63	21.50%
	Ph.D.	31	10.58%
